# Synergistic Antimicrobial Action of Chlorhexidine and Ozone in Endodontic Treatment

**DOI:** 10.1155/2014/592423

**Published:** 2014-05-28

**Authors:** Rita Noites, Cidália Pina-Vaz, Rita Rocha, Manuel Fontes Carvalho, Acácio Gonçalves, Irene Pina-vaz

**Affiliations:** ^1^Department of Endodontics, Catholic University of Portugal, Campus Viseu, Estrada da Circunvalação, 3504-505 Viseu, Portugal; ^2^Department of Microbiology, Faculty of Medicine, Alameda Professor Hernâni Monteiro, 4200-319 Porto, Portugal; ^3^Department of Endodontics, Faculty of Dentistry, Rua Dr. Manuel Pereira da Silva, 4200-393 Porto, Portugal

## Abstract

*Objectives*. The aim of this study was to determine whether irrigation with sodium hypochlorite, chlorhexidine, and ozone gas, alone or in combination, were effective against *Enterococcus faecalis* and *Candida albicans*; these are microorganisms frequently isolated from teeth with periapical lesions resistant to endodontic treatment. *Material and Methods*. 220 single root teeth, recently extracted, were inoculated with *Candida albicans* and *Enterococcus faecalis*. The formulations tested were sodium hypochlorite at 1, 3, and 5% chlorhexidine at 0.2% and 2% and ozone gas applied for different periods of time. The combination of sodium hypochlorite at 5% and chlorhexidine at 2%, with gaseous ozone, were also assessed. For the most active treatments the mechanism of action was assessed through flow cytometry. *Results*. Sodium hypochlorite, chlorhexidine, and gaseous ozone alone were ineffective in completely eliminating the microorganisms. The association of chlorhexidine at 2% followed by ozone gas for 24 seconds promoted the complete elimination of *Candida albicans* and *Enterococcus faecalis*. Flow cytometry shows that ozone and chlorhexidine act differently, which could explain its synergic activity. *Conclusions*. This new disinfection protocol, combining irrigation with chlorhexidine at 2% and ozone gas for 24 seconds, may be advantageous when treating infected root canals.

## 1. Introduction 

Many studies [1–6] have clearly demonstrated the major role of the microorganisms in the etiopatogeny of pulpar and periapical lesions. Elimination of microorganisms and their by-products from the root canal system is a main goal of endodontic treatment, though not always achieved [7, 8]. Secondary infection (after treatment) is caused by microorganisms that resisted intracanal antimicrobial procedures and periods of deprivation, in treated canals [9, 10].* Enterococcus faecalis* (*E. faecalis*), a persistent organism that can survive as a monoculture in root canals [9, 10], and the yeast* Candida albicans* (*C. albicans*) are frequently recovered from root-filled teeth with persistent periapical lesions [6, 8, 11]. Although techniques and materials have improved along time, the success rates of these treatments do not appear to have improved much over the last century [12].

Although sodium hypochlorite is the most commonly used irrigant, chlorhexidine has recently been introduced as an alternative irrigating solution. However, it does not described any possible effect of the association between them.

Chlorhexidine is a cationic molecule, which can be used during treatment. It has a wide range of antimicrobial activity. Furthermore, because of its cationic structure, chlorhexidine has a unique property named substantivity [13].

Ozone is being presented as a possible alternative antiseptic agent due to its antimicrobial power [14] and low cytotoxicity [15]; however, there is little information regarding the time and concentration to use.

Flow cytometry has been proven as an excellent tool in microbiology field to quickly demonstrate antimicrobial activity as well as to clarify about mechanism of action/resistance [16].

Our study purposes a novel approach to be used in endodontic treatment, leveraging the synergy achieved between two products with antibacterial action which is effective against* E. faecalis* and* C. albicans*.

## 2. Materials and Methods

### 2.1. Selection and Preparation of the Teeth

220 extracted human single rooted teeth were selected to this study. Working length was determined using a size 15 K-file (Maillefer Inc.), until its tip became visible from the apical foramen, 1 mm short of the apical foramen. Teeth were prepared up to a size 40 K-file (Maillefer Inc.). Teeth were irrigated with 2 mL of saline solution at each change of file. Smear layer was removed with 10% acid citric solution (10 mL) and a final irrigation of saline solution was made according to the method of Schäfer and Bössmann [17]. The apexes were sealed with composite. Two coats of nail varnish were applied to the external surface of the roots. The teeth were then sterilized with plasma hydrogen peroxide (Sterrad ASP).

### 2.2. Root Canal Contamination

*E. faecalis* ATCC2912 and* C. albicans* ATCC90028 were cultured for 24 hours at 37°C in Brain-Heart agar (Liofilchem, Italy) and Sabouraud dextrose agar (Liofilchem, Italy), respectively. A cell suspension of 1 × 10^8^ cells/mL was prepared in Brain-Heart infusion broth (Liofilchem, Italy) for each microorganism. Subsequently, the teeth were incubated aerobically at 37°C for 3 days. The inoculum was refreshed after 48 hours.

### 2.3. Efficacy of the Treatments on Contaminated Root Canal

The teeth were randomly assigned to four experimental groups for* E. faecalis* or* C. albicans*, subjected to different treatments.

In the control group, the contaminated root canals of 10 teeth were irrigated with 10 mL of 0.9% (w/v) sterile saline solution.

In the sodium hypochlorite (NaOCl) group, teeth were divided into three subgroups of 10 teeth each. Root canals were irrigated with 10 mL of 1%, 3%, or 5% NaOCl solution.

In the chlorhexidine (CHx) group, teeth were divided into two subgroups of 10 teeth each. Root canals were irrigated with 10 mL of 0.2% or 2% CHx solution.

In the ozone group, teeth were divided into four subgroups of 10 teeth each. Gaseous ozone was applied with an ozone generator (Prozone, W&H) for 24, 60, 120, and 180 seconds.

Upon treatment, all teeth were washed with 10 mL of sterile saline solution. In order to assess the number of viable microorganisms, the root canals were incubated with Brain-Heart infusion broth at 37°C for 24 h, because if smeared immediately no growth was visible. Viable microorganisms were quantified by determining the number of colony-forming units (CFU) in Brain-Heart agar or Sabouraud dextrose agar for* E. faecalis* and* C. albicans*, respectively.

### 2.4. Combinatorial Effect of Gaseous Ozone with Sodium Hypochlorite or Chlorhexidine

The association between NaOCl or chlorhexidine and ozone was evaluated in 20 teeth (10 infected with* E. faecalis* and 10 infected with* C. albicans*). Thus, root canals were irrigated with 10 mL of 5% NaOCl solution or 2% CHx followed by the application of gaseous ozone for 24, 60, 120, and 180 seconds. CFUs were determined as described above.

### 2.5. Clarification of Mechanism of Action

In order to clarify the mechanisms of action of CHx and ozone, the most effective agents, the following different fluorescent markers were used: propidium iodide (PI) binds to DNA but only if cell cytoplasmatic membrane is damaged, that is, death (Molecular Probes Europe BV, Leiden, Netherlands) and bis-(1,3-dibutylbarbituric acid) trimethine oxonol (BOX) is a lipophilic and anionic fluorescent stain that accumulates intracellular when the cytoplasmatic membrane is depolarized (Molecular probes) and additionally only regarding yeast FUN-1, a fluorescent probe that is converted only by metabolically active cells. Acquisition was performed with cellQuest TM pro Software and based on light-scatter and fluorescence signals resulting from 15 mW laser illumination at 488 nm and 635 nm. Signals corresponding to forward and side scatter (FSC and SSC) and fluorescence were accumulated at FLI (530/30 nm) for DIBAC, FL2 (620 nm) for FUN-1 and FL3 (>670 nm) for PI. For sample preparation, 1 × 10^6^ cells/mL of each strain was incubated with each fluorescent probe for 30 min in the dark at 1 *μ*g/mL for DIBAC and PI and 0.5 *μ*M of FUN-1. A control suspension, not exposed to any treatment or staining, was used as autofluorescence and others stained with the different probes were used as viable control. At least 30 000 cells were analyzed on a BD Biosciences FACSCalibur.

*E. faecalis* and* C. albicans* were treated for 24 s, 60 s, 120 s, and 180 s with ozone and with 0.2% and 2% of CHx for 1, 2, 3, and 4 minutes and stained with different fluorochromes, in order to understand its primary mechanism of action that could explain the synergic effect.

### 2.6. Data Analysis

Statistical analysis was performed using a* Statistical Package for Social Sciences* (SPSS) version 19.0 (IBM, Armonk, New York, USA).

In the description of the database the percentages (%) and absolute value (N) were used, since the dependent variables were dichotomous (presence or absence of microorganisms and effectiveness or ineffectiveness of the formulations tested). The comparative analysis of different irrigation solutions and between different concentrations within the same solution was performed using the chi-square test. The significance level (*α*) to reject the null hypothesis was set at 0.05, with a confidence interval (CI) of 95%.

## 3. Results

### 3.1. Efficacy of Root Canal Treatments

**Figure 1:**
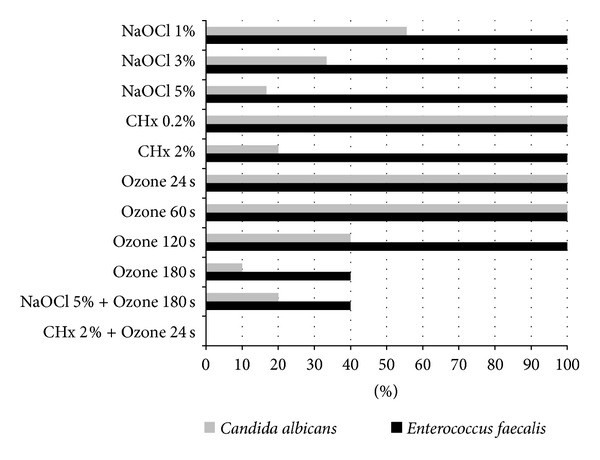
Percentage of teeth failed after different treatments.

All the tested concentrations of NaOCl were ineffective to completely disinfect the roots, being the percentage of failure teeth inversely related to the concentration. Regarding* C. albicans* 5% NaOCl was significantly more effective than 1% (*χ* ^2^ = 4.0, *P* < 0.05) (Figure 1), while 3% NaOCl was not significantly better than 1% NaOCl (*χ* ^2^ = 0.9, *P* = 0.343). The success rate for* E. faecalis* eradication was only 9% (91% of failure teeth) even with 5% NaOCl. Regarding the irrigation with CHx, the concentration of 0.2% had minimal antimicrobial activity against both microorganisms. 2% CHx was very efficient for* C. albicans*, being the success rate of 90%, which was significantly better than 0.2% (*P* < 0.001). For* E. faecalis* 2% CHx was totally inefficient (Figure 1). The application of gaseous ozone during short periods (24 and 60 seconds) was not sufficient to eliminate neither* C. albicans* nor* E. faecalis* (Figure 1). Higher periods (120 s and 180 s), although not completely efficient, were significantly better than lower doses (*χ* ^2^ = 16.58; *P* = 0.001, for* C. albicans* and *χ* ^2^ = 10.58; *P* = 0.014, for* E. faecalis*).

### 3.2. Combinatorial Effect of Gaseous Ozone with Sodium Hypochlorite or Chlorhexidine

The 5% NaOCl combined with ozone, even in the maximum time (180 s), did not show significant differences from the isolated treatments (Figure 1). Nevertheless, the results indicate a great antimicrobial activity with 2% CHx followed by gaseous ozone even only for 24 s (Figure 1), with a complete elimination of both* C. albicans* and* E. faecalis*.

### 3.3. Mechanism of Action

**Figure 2:**
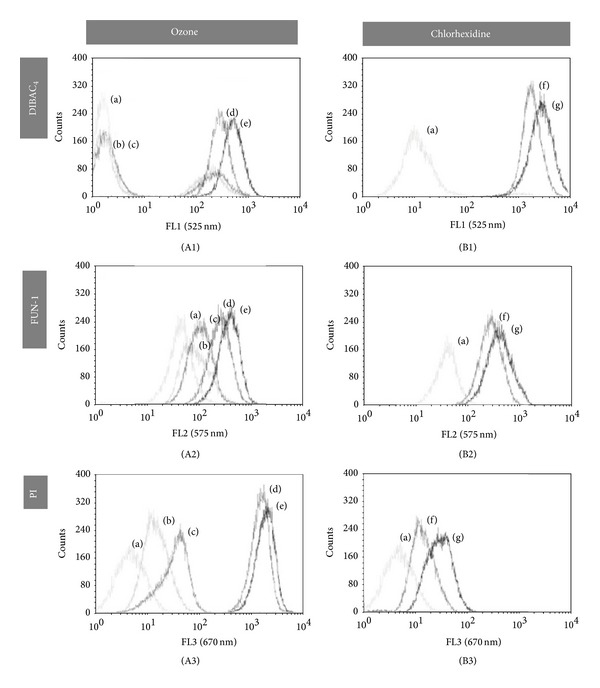
*In vitro* evaluation of antimicrobial activity of ozone (A) and chlorhexidine (B) against* C. albicans* by flow cytometry. Distribution of fluorescence intensity from yeast cells treated with ozone (A) for 30, 60, 120, and 180 seconds ((b), (c), (d), and (e), resp.), stained with DiBAC_4_ (A1), FUN-1 (A2), and PI (A3); and cells treated with 0.2 (f) and 2% (g) of chlorhexidine (B) for 3 minutes, stained with DiBAC_4_ (B1), FUN-1 (B2) and PI (B3). Fluorescence of untreated cells stained with fluorescence marker is represented by (a).

**Figure 3:**
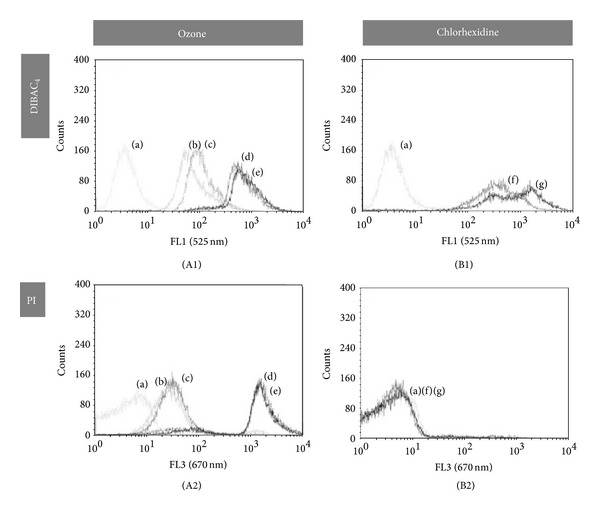
*In vitro* evaluation of antimicrobial activity of ozone (A) and chlorhexidine (B) against* E. faecalis* by flow cytometry. Distribution of fluorescence intensity from bacterial cells treated with ozone (A) for 24, 60, 120, and 180 seconds ((b), (c), (d), and (e), resp.), stained with DiBAC_4_ (A1) and PI (A2); and cells treated with 0.2 (f) and 2% (g) of chlorhexidine (B) for 3 minutes, stained with DiBAC_4_ (B1) and PI (B2). Fluorescence of untreated cells stained with fluorescent marker is represented by (a).

*E. faecalis* and* C. albicans* were permeable to PI after 120 or 180 s of ozone but not after CHx, even at 2% for 4 minutes (Figures 2 and 3). BOX staining showed an increase of fluorescence after both treatments in a dose/time-dependent manner for both microorganisms (Figures 2 and 3). FUN-1 staining increased after both treatments regarding* C. albicans* (Figure 3).

## 4. Discussion 

The main findings of the present study indicate that the irrigation with 2% CHx and 24 s of gaseous ozone may be advantageous, particularly for postdisease treatment. This protocol involving the synergism between ozone gas and 2% CHx solution, never described before, seems to be effective and has potential to be used in the clinical practice. Furthermore, it is one of few protocols described with complete elimination of the microorganisms usually resistant to endodontic treatment, particularly in the tooth model, contaminated with a pure culture. In addition, flow citometry is shown to be an excellent tool to clarify the antimicrobial effect of the drugs [16].

In the present study the irrigating solutions tested, hypochlorite and CHx, showed antimicrobial activity particularly on* C. albicans*. Regarding different concentrations, sodium hypochlorite 5% was significantly more effective than 1%. The success rate for* E. faecalis* eradication was much lower, even with sodium hypochlorite at 5%.

Similarly, 2% CHx was very efficient for* C. albicans* and almost ineffective for* E. faecalis*. Yet, even the more efficient irrigating solutions were not able to completely inhibit the microorganisms studied.

10% citric acid solution was used to finish the chemomechanical preparation in order to remove the smear layer formed during the root canal preparation [18].

The application of gaseous ozone in short periods of time as 24 s and 60 s, was not completely effective for any of the microorganisms.

Previous studies showed similar results. Gomes et al. 2001 [19] testing various concentrations of NaOCl and CHx during different periods, in cell suspensions of* E. faecalis*, found that even though all tested irrigants were effective in killing* E. faecalis*, the time required depended on the concentration and type of irrigant used. In this study [19] the antimicrobial activity of CHx and NaOCl took place through contact with* E. faecalis* suspension. In our study we used the infected tooth model which can explain less activity of the antimicrobial substances, compared with studies of incubation of broth cultures [19, 20]. Nevertheless, the tooth model is a way of simulating the infection “*in vivo*.” The different methodologies can explain different results. The irrigants directly in contact with the bacterial cells exert an action immediately, as in root canals the direct contact between bacteria and irrigants may be prevented.

*E. faecalis* and* C. albicans* are two well-known resistant strains implicated in periapical lesions resistant to endodontic treatment. In that sense they have been used in many studies about antibacterial efficacy of root canal irrigants or intracanal dressings and were included here.

Ozone is being presented as a possible alternative antiseptic agent due to its antimicrobial power [15, 21] and low cytotoxicity [15]. However, there is little information regarding the time and concentration to use. In this study, the ozone gas alone, applied during short periods (24 s and 60 s), showed no efficacy on any of the antimicrobial agents tested. Regarding* C. albicans* ozone gas for 180 seconds had an activity significantly higher compared to shorter times. For* E. faecalis* greater exposure times of ozone were not completely effective but showed a greater activity than that obtained by NaOCl or CHx in any of the concentrations. Similarly, Estrela et al. [21] indicated that the application of ozone gas for 20 minutes in infected human root canals was not sufficient to inactivate* E. faecalis*. Hems et al. [22] found significant reduction in bacterial cell numbers but only detected after 240 s of application. The duration of action can be, therefore, an important consideration in ozone antibacterial effect.

In the present study, even with the highest periods (120 s, 180 s), ozone was not completely efficient.

The possible synergism between ozone gas and the irrigating solutions of sodium hypochlorite 5% and 2% CHx was investigated. These solutions were selected because they were the ones that had better antimicrobial activity, by themselves. A complete elimination of both* C. albicans* and* E. faecalis* was obtained with 2% CHx and 24 s of gas ozone.

The synergism obtained can be explained by the distinct mode of action. Both ozone and chlorhexidine depolarize the cells in dose-dependent manner.

In conclusion, only the combined action of 2% CHx and ozone gas for short period promotes the complete elimination of both microorganisms tested in the tooth model. The results of this study could lead to redirect efforts towards new protocols for reducing microbial load in infected root canals looking for synergisms between new or already known antimicrobial products.
